# Burnout Syndrome among Emergency Department Staff: Prevalence and Associated Factors

**DOI:** 10.1155/2019/6462472

**Published:** 2019-01-21

**Authors:** Audrey Moukarzel, Pierre Michelet, Anne-Claire Durand, Mustapha Sebbane, Stéphane Bourgeois, Thibaut Markarian, Catherine Bompard, Stéphanie Gentile

**Affiliations:** ^1^Service d'Accueil des Urgences, Hôpital de la Timone 2, Assistance Publique des Hôpitaux de Marseille, 264 rue Saint Pierre, 13385 Marseille Cedex 5, France; ^2^Equipe de Recherche EA 3279 “Santé Publique, Maladies Chroniques et Qualité de Vie”, Faculté de Médecine, Aix-Marseille Université, 27 boulevard Jean Moulin, 13 385 Marseille Cedex 5, France; ^3^Département de Médecine d'Urgence, CHU Lapeyronie, Université Montpellier 1, 34295 Montpellier, France; ^4^Service d'Accueil des Urgences, Centre Hospitalier Henri-Duffaut, 305 rue Raoul Follereau, 84000 Avignon, France; ^5^Service d'Evaluation Médicale, Hôpital de La Conception, Assistance Publique des Hôpitaux de Marseille, 147 boulevard Baille, 13005 Marseille, France

## Abstract

**Objectives:**

Emergency department (ED) professionals are exposed to burnout syndrome due to excessive workload and high demands for care. The objective of our study was to assess the prevalence burnout among all ED staff and to determine associated factors.

**Methods:**

A cross-sectional survey was conducted in 3 EDs. The data were collected using a standardized questionnaire. It included demographical and occupational data, general health questions, burnout level (Maslach Burnout Inventory), job strain (Karasek), and quality of life (Medical Outcome Study Short Form).

**Results:**

Of the 529 professionals working in EDs, 379 responses were collected (participation rate of 71.6%). Emotional exhaustion (EE) and depersonalization (DP), the major components of burnout, were reported, respectively, by 15.8% and 29.6% of the professionals. Burnout prevalence was 34.6%, defined as a severely abnormal level of either EE or DP. The medical category was significantly more affected by the burnout compared with their colleagues: nearly one ED physician out of two had a burnout (50.7%). In the multivariate analysis of covariance, job strain and a low mental component score were the two main factors independently associated with burnout (p < 0.05).

**Conclusion:**

The results of our study show that ED professionals are a vulnerable group. Preventive approaches to stress and burnout are needed to promote quality of work life.

## 1. Introduction

Changes in working conditions are associated with a growing prevalence of psychosocial risks that could affect physical and mental health [1–5]. After musculoskeletal disorders, work-related mental suffering is the second most common work-related condition described in the active employee population [6]. Burnout syndrome accounted for 7% of the psychological disorders reported by occupational physicians in France in 2012. If an extrapolation is made of the 480,000 employees potentially affected by work-related mental suffering, this would represent about 30,000 cases [1].

According to the European Agency for Safety and Health at Work, France appears to be behind its neighbours in the efforts of companies to combat stress at work and prevent psychosocial risks [7]. Awareness only began to rise in the 2000s with cases of psychological harassment [8] and following a wave of suicides in the companies* France Télécom* and* Renault*. Following these events, a number of regulatory measures were introduced, such as the law punishing psychological harassment at work in 2002 and an emergency plan for the prevention of psychosocial risks [9] by the Minister of Labour in 2009.

It was only in 2016, as part of the third Occupational Health Plan [10], that the Minister of Health referred the matter to the High Authority of Health (HAS). It published a report in March 2017 in which it proposed a definition of burnout and recommendations for good practice for occupational and general practitioners [11].

At the same time, the HAS has been working since 2010 on the concept of quality of life at work as part of the certification of health care institutions. It has demonstrated the impact of poor quality of life in the workplace on patient safety [12]: the health sector is thus particularly affected by increasingly difficult working conditions, mainly in the emergency departments (ED) [12–21]. Indeed, EDs suffer from an ever-increasing demand for care [16], difficulties in exercising due to the lack of downstream structures, and an increasing complexity of care, particularly with the increase in the number of elderly patients [16–20].

Few studies in France have analyzed burnout and work stress among ED professionals [19, 20, 22–24]. The only national study conducted in 2012 targeted only the medical profession [22]. Other French studies have been published on the topic of emergency but they also targeted intensive care or the Emergency Medical Assistance Service (SAMU) [19, 20, 23, 24].

The aims of our study were to assess the prevalence of burnout among all staff working in EDs in three French University Hospital, and to determine associated factors. This is the first study in France, to our knowledge, targeting all professionals.

## 2. Materials and Methods

### 2.1. Study Design and Population

A cross-sectional study was conducted from September to November 2016 in three EDs located in the South France:

- The Timone University Hospital of Marseille.
- The Henri-Duffaut Hospital of Avignon.
- The Lapeyronie University Hospital of Montpellier.

All professionals working in the three EDs were included in the study. Three professional categories were involved: medical, paramedical (nurses, health managers, and health care assistants), and administrative and technical (administrative reception agents, hospital service agents, social workers, psychologists, mediators, and secretaries). Excluded were students or trainees as well as professionals working only in the Emergency Medical Assistance Service (SAMU).

### 2.2. Description of the Survey and Measures

The survey was strictly confidential and anonymous in accordance with the law of 6 January 1978 on data processing [25]. Prior to the start of the survey, all professionals in the three EDS were informed of the objectives and interests of the study and their right to refuse or agree to participate. Their verbal consent was obtained.

Data were collecting using a standardized self-administered questionnaire divided into four sections. The first section covered sociodemographic data (age, sex, and family situation) and professional characteristics (professional activity, time working in the profession, work rhythm, and the intention to quit the ED), physical activity and lifestyle (sport, consumption of tobacco, alcohol, and coffee), and work-related health (at least one sick leave in the last 12 months and sleep disorders).

The second part assessed job strain by the French version of the Karasek [26]. It consists of 26 questions and assesses three dimensions: the psychological demand to which an employee is subjected, the decision-making latitude granted to him and the social support he receives from the hierarchy and his colleagues. The psychological demand and decision latitude dimensions were divided into median based cut-points. Four possible combinations (groups) of job strain variables were created: (a) low strain (low demands (below median) combined with high decision latitude (above median)); (b) active (high demands (above median) and high decision latitude (above median)); (c) passive (low demands (below median) and low decision latitude (below median)); and (d) high strain or job strain (high demands (above median) and low decision latitude (below median)). In this analysis, all participants are grouped into one of the four categories (job strain, low strain, passive, and active categories) and we limited our measure to job strain.

The third section assessed the quality of life using the Medical Outcome Study Short Form in 12 items or SF-12 [27]. This is a shortened version of the SF-36 evaluating eight dimensions: physical functioning, physical limitation, physical pain, general health, vitality, social functioning, emotional limitation, and mental health. These were summarized into two scales: a physical component score (PCS) and a mental component score (MCS), in accordance with the guidelines for the SF-12 instrument [28]. Both scores ranged between 0 and 100, with a higher score indicating better health. These SF12-based summaries have been shown to reproduce accurately both the PCS and the MCS derived from the full SF-36 [29].

The last section evaluated burnout syndrome using the French version of the Maslach Burnout Inventory (MBI) questionnaire [30]. It is a 22-item questionnaire and explores the three dimensions of burnout: emotional exhaustion (EE; 9 items), depersonalization (DP; 5 items) or dehumanization, and personal achievement (PA; 8 items). The total score of each dimension is classified as low, moderate or high according to the cut-offs published by Maslach and Jackson [31]. In our study, respondents with high levels of either EE or DP were defined as having burnout. Among them, those with low personal achievement were considered with high burnout. The lowest scores indicated the absence of the syndrome. Most studies consider EE and DP to be the fundamental dimensions of burnout, while a low level of personal achievement would appear to develop in parallel [11, 13]. We therefore limited our measure of burnout to EE and DP.

### 2.3. Statistical Analysis

The data collected were analyzed using the SPSS software version 20.0. In a first step, a univariate analysis was conducted to characterize the professional categories of EDs. The associations between qualitative variables were measured by Chi-square test and the exact Fischer test for small numbers. Analysis of variance (ANOVA) was used to compare quantitativevariables between the three professional categories. A p-value of < 0.05 was considered statistically significant.

We performed a stepwise linear regression to assess independent association between variables and the two burnout subscores. All covariates with p-value ≤ 0.1 in the univariate linear regression were included in the multivariate analysis. We performed a backward stepping procedure

## 3. Results

During the survey period, there were 529 professionals working in the three EDs. Of these, 379 completed the questionnaire, corresponding to a response rate of 71.6%. Response rates were higher in the EDs of Marseille and Avignon (more than 80%) than in the ED of Montpellier (52%). The proportion of nonrespondents was similar regardless of the professional category.

Of the 379 respondents, the ED in Marseille represented 45.1% of the professionals surveyed, the ED in Avignon 30.1%, and the ED in Montpellier 24.8%.

### 3.1. Population Characteristics by Occupational Category

Table 1 presents characteristics among the respondents overall and between the three occupational categories.

The population interviewed was composed of 18.2% physicians, 70.7% paramedics and 11.1% administrative and technical (A/T) staff. Twenty-six percent of the respondents were men. Men were more numerous in Montpellier University Hospital (40.9%) than in the other two EDs (20.2% in Avignon University Hospital and 21.8% in Marseille, p = 0.001). The average age of the professionals was 37.4 ± 9.9 years with ages ranging from 22 to 65 years. The A/T category was the oldest (p < 0.02), but also more female (p < 0.001) than that of physicians and paramedics.

Almost two-thirds of professionals had a night activity (59.8%), either only at night or both day and night. For almost 70% of the respondents, this was their first job within an ED, particularly for the A/T (80.5%) and paramedical (70.5%, p = 0.02) categories, regardless of the ED.

The paramedical and A/T categories reported twice as many sick leaves as physicians (49.1% and 50.0% respectively, versus 19.1% for physicians, p < 0.001).

Smokers represented 38.3% of the professionals surveyed; the A/T category was the most concerned (45.2%, p = 0.038). Physicians were the most frequent coffee drinkers: 29.4% reported drinking more than 4 cups of coffee per day (p = 0.042). Very few professionals in the study declared that they consumed alcoholic beverages on a daily basis (0.8%). A third had poor sleep quality (35.5%) and more than half practiced a sporting activity at least once a week (57%), particularly professionals from the EDs of Montpellier and Avignon (68.9% and 60.5%, compared to 48.2% for Marseille, p < 0.02).

Finally, 17.1% of professionals had expressed a desire to quit the ED, with no significant difference according to professional category and emergency department.

### 3.2. Burnout Levels and the Desire to Quit the ED

Overall, 132 ED professionals (34.6%) reported a severely high score for either EE or DP, thus meeting the burnout criteria (Table 1). Moreover, 15.8%, 29.6%, and 41.4% of respondents exhibited high emotional exhaustion, high depersonalization, and a low sense of personal accomplishment, respectively. The medical category was the category most affected by burnout: 50.7% compared to 32.1% for paramedics and 23.4% for A/T (p < 0.004).

ED physicians were the most affected by a high level of DP (p = 0.001) and by a low level of PA (p = 0.010). There was no significant difference between the three categories and a high level of EP. Professionals working in the ED of Marseille were more affected by burnout (42.7%) than those in the other two EDs (26.3% in Avignon University Hospital and 29.8% in Montpellier, p = 0.009). The ED of Marseille was more affected by DP (p = 0.003), there was no significant difference between the other dimensions and the three EDs.

Seventeen percent of the ED professionals wanted to quit the ED without difference between occupational categories.

### 3.3. Job Strain

Out of the respondents, 30.1% of them were experiencing the job strain with no significant difference between the EDs. The two categories A/T and paramedical were the most concerned by the job strain (p < 0.001), due in particular to a significant lack of decision-making latitude for A/Ts (78.6%) and low social support at work for paramedics (66.8%). Physicians were the professionals who suffered the most from a high level of psychological demand (79.7%, p < 0.02).

### 3.4. Quality of Life of ED Professionals

The average physical and mental component scores of ED professionals were 48.2 ± 6.0 and 39.6 ± 9.9 points, respectively. ED physicians have lower MCS than the other two categories (-4.4 ± 1.2 points compared to paramedics, p°<°0.001; and -4.6 ± 1.8 points compared to A/T, p = 0.029). However, no significant difference was observed between PCS and occupational categories.

### 3.5. Factors Associated with Burnout

Table 2 shows the results of the univariate linear regression of MBI emotional exhaustion depersonalization and personal achievement scores with relevant covariate measured in the study. EE, DP, and PA were more frequent among ED professionals working at night, experiencing job strain, sleep disorders, and who having a lower MCS (p < 0.05). In addition, medical category was the most affected by EE and DP (p < 0.05). The desire to quit the ED was strongly associated with higher EE and DP and lower PA scores (p < 0.001).

In the multivariate analysis of covariance, job strain and a low MCS were the two main factors independently associated with both EE, DP, and PA scores (p < 0.05). In addition, medical category was associated with both EE and DP scores (p< 0.05), as shown in Table 3.

## 4. Discussion

This is the first French study that included all permanent professional working in three emergency departments, namely, physicians, paramedics and administrative/support staff (response rate 71.6%). Our results show that significant burnout was reported by 34.6% of respondents, and that it was mainly associated with two factors: job strain and low MCS. It was also more pronounced for the medical category.

Before discussing our results of the burnout and associated factors, we would like to highlight some methodological remarks. First, in the previous studies on burnout, the prevalence of burnout is always not specified. Indeed, the authors often described the results for each dimension, without combining them to obtain the prevalence [32–39]. Second, the method to identify burnout stages is not always identical. The authors often presented compiled results, without detailing the different dimensions of the MBI, which prevented the comparisons of burnout level with certain studies [32, 40]. Third, most of the studies, in our field of research, did not only target ED professionals but also professionals of intensive care unit [23, 24, 39]. Moreover, in most studies burnout was examined among only one professional category: nurses or physicians [32, 37, 41]. At last, most studies presented a low response rate which produces a selection bias [15, 22–24, 41].

Our study highlighted that the medical category was significantly more affected by the burnout compared with their colleagues. In fact, nearly one ED physician out of two had a burnout (50.7%). This result is very evident in the multivariate analysis, ED physicians exhibited higher levels of EE and DP. This result is consistent with the great majority of published studies, regardless of the country, which have shown burnout prevalence rates among ED physicians ranging from 11% [22] to 71.4% [15, 32, 42, 43]. This rate is higher than the only French study conducted by Sende et al. more than 10 years ago [22].

This difference raises questions about the evolution of working conditions, which have most certainly deteriorated since 2006 (year of collection of the national study carried out by Sende et al.) and 2016 (date of our collection), as can be observed in many press articles [44, 45].

Fewer ED physicians have a high level of emotional exhaustion: 21.7% versus 29% to 71% in the other studies [15, 22, 33, 35, 37, 38, 43]. It may result from the progressive loss of the health care worker's ability to feel emotionally involved in their work. Our hypothesis is that the practice of emergency medicine leaves little space for emotions. Indeed, in response to the patient's condition, it is necessary to act quickly and effectively. In this context, physicians do not allow themselves to be invaded by their emotions. This translates in keeping distance with the patient: work on one side and feelings on the other side, as also shown by Lloyd and Laurent [19, 20, 38]. In contrast, our physicians were twice as likely (55.1%) to have low levels of PA. This is reflected in a negative assessment of their work and skills, a perception that the objectives are not being achieved, and a decrease in self-esteem and a sense of self-efficacy [46].

The professionals in the EDs in our survey, and particularly physicians, have a lower quality of life in both dimensions, physical and mental, than that of the general French population [27]. Indeed, the physical and mental quality of life scores of ED professionals were 4.7 points and 8.8 points lower respectively than those of the French population (p < 0.001). This result is consistent with other studies that have shown that health professions have lower levels of quality of life [47, 48]. The comparison with a Swiss study conducted by Bovier et al. [49] and carried out on a sample of nonemergency physicians, shows that our physicians have much lower quality of life scores: 49.7 versus 53.5 points for the physical score and 36.6 versus 48 points for the mental score.

In our study, the paramedical category is also affected by burnout with a prevalence of 32.1%, which is lower than that of physicians. This rate is similar than what is found in the literature (ranging from an average of 25% in the meta-analysis of Adriaenssens et al. [21] to 40% for nurses in the European PRESST-NEXT study [42]).

Professionals in the 3 EDs are also affected by job strain: 30.1% felt that their work was very stressful, i.e., nearly one in three professionals. Contrary to the burnout results, the paramedical and administrative and technical categories were the most impacted with 2.5 and 3.8 times more risk of being tense at work respectively. Compared to the general French employed population, our result is higher (30.1% versus 24% in the SUMMER survey) [50]. However, the proportion of professionals in job strain in our study is in the low range of rates found in the literature on EDs, which vary from 24% to 60.7% [36, 37, 51, 52].

The two main factors associated with burnout were job strain and low MCS. The emergency service cumulates many characteristics of a stressful job: heavy workload, working in emergency, zapping, “always more and always better” with constant resources, uncertainty, lack of recognition and frustration, interpersonal conflicts, just-in-time production, from incivility to environmental violence [22]. Thus, in our study burnout is associated with the desire to quit the ED and 17.1% of ED professionals expressed it.

The main limit of our study is the study design. Indeed, the cross-sectional nature of the study imposes temporal limitations. It cannot prove cause-and-effect relationship between associated variables, particularly about the health of ED professionals. Thus, we could not assess whether, for example, a level of burnout is a result of a sleep disorders, or is it a cause. A cohort study or panel study may allow us to analyze risk factors and use correlations to determine absolute predictive factors. However, this study provided a first measure of the prevalence of burnout among a group of professionals working in three French emergency departments and updated data on ED physicians. It presents a good participation rate (71.6% versus 57% on average [22]) and was based on questionnaires validated and recognized by the scientific community [26, 27, 30, 31]. It highlights the serious problem of the suffering of ED professionals by showing a risk of burnout. It objectifies, through scientific data, the various examples of suffering shown in the news. These results should be taken into consideration for the health of professionals and the improvement in the quality of care. Indeed, as Westbrook et al. [53] demonstrate, quality of work life can lead to medical errors with repercussions such as compromised health system performance. Perhaps the health of our professionals should be considered as an important determinant of quality of care and patient health, or is it still a “forgotten” indicator of the quality of our systems as Wallace suggests [54].

## 5. Conclusion

The results of our study show that ED professionals are a vulnerable group since almost half of them have experienced burnout. Indeed, they are confronted with intense and repetitive situations that are specific to emergency profession (severity of pathologies, unpredictability of situations, emotional load, and frequent physical and verbal violence) which constitute a favorable ground for the development of stress and burnout. Constraints related to the work environment, such as mission requirements, restructuring, and return to balance, are also factors that affect the mental health of professionals. One of the major challenges for institutions is to identify these tense situations, which are very damaging to the health and productivity of their staff. This study shows the need for preventive approaches to stress and burnout, particularly in emergency medicine, which must be anticipated to promote the quality of work life.

## Figures and Tables

**Table 1:** Population characteristics amongst the respondents overall and between the three occupational categories.

		**Overall**	**Physicians**	**Paramedical**	**A/T**	**p**
		n = 379	n = 69	n = 268	n = 42	

Demographics	Age (n=373)	37,4 ± 9,9	37,3 ± 8.9	36,8 ± 10,0	42,4 ± 10,8	0,003
	Women (n=377)	279 (74,0)	39 (56,5)	203 (76,3)	37 (88,1)	< 0,001
	Live in couple (n=376)	265 (70,5)	53 (76,8)	184 (69,2)	28 (68,3)	0,440
	Have children (n=375)	196 (52,3)	34 (49,3)	140 (52,8)	22 (53,7)	0,855

Professional characteristics	1^st^ job in a ED (n=378)	261 (69,0)	39 (56,5)	189 (70,5)	33 (80,5)	0,020
	Time working in the ED: > 5 years (n=379)	195 (51,5)	36 (52,2)	135 (50,4)	24 (57,1)	0,710
	Work rythm (Night or Day/Night) (n=376)	225 (59,8)	66 (95,7)	144 (54,1)	15 (36,6)	< 0,001
	Wish to change ED (n=346)	59 (17,1)	10 (15,9)	43 (17,6)	6 (15,8)	0,929

work-related health	≥1 sick leave during the last year (n=371)	162 (43,7)	13 (19,1)	130 (49,1)	19 (50,0)	< 0,001
	Sleep disorders (n=375)	153 (41.9)	31 (46.3)	111 (42.9)	11 (28.2)	0.163

Lifestyle	Regular sport activity (n=372)	212 (57,0)	39 (58,2)	152 (57,4)	21 (52,5)	0,825
	Smoker (n=376)	144 (38,3)	17 (25,0)	108 (40,6)	19 (45,2)	0,038
	> 4 cups of coffee per day (n=375)	70 (18,7)	20 (29,4)	43 (16,2)	7 (17,1)	0,042
	Daily consumption of alcohol (n=374)	3 (0,8)	2 (2,9)	-	1 (2,4)	0,025

Quality of life (SF-12)	PCS	48,2 ± 6,0	49,7 ± 4,9	47,8 ± 6,2	48,2 ± 7,4	0.070
	MCS	39,6 ± 9,9	36,6 ± 10,4	40,0 ± 9,9	41,8 ± 8,1	0.012

Tense at work (Karasek)	High level of psychological Demand	234 (61,7)	55 (79,7)	157 (58,6)	22 (52,4)	0,002
	Low level of decisional Latitude	210 (55,4)	14 (20,3)	163 (60,8)	33 (78,6)	< 0,001
	Low level of social Support at Work	255 (67,3)	44 (63,8)	179 (66,8)	32 (76,2)	0,381
	Job strain (n=379)	114 (30.1)	12 (17.2)	86 (32.1)	16 (38.1)	0.029

MBI	Burnout (n=379)	132 (34.6)	35 (50.7)	86 (32.1)	11 (23.8)	0.004
	High level of EE (score ≥ 30)	60 (15.8)	15 (21.7)	43 (16.0)	2 (4.8)	0.057
	High level of DP (score ≥ 12)	112 (29.6)	32 (46.4)	72 (26.9)	8 (19.0)	0.001
	Low level of PA (Score ≥ 40)	157 (41.4)	38 (55.1)	98 (36.6)	21 (50.0)	0.010

Data are mean±standard deviation or frequency (percentage).

**Table 2:** Univariate linear regression of MBI emotional exhaustion, depersonalization, and personal achievement with relevant covariates.

**Category**	**Covariate**	**EE**		**DP**		**PA**	

		Coefficient [SD]	p	Coefficient [SD]	p	Coefficient [SD]	p

Demographics	Age (years)	-0.03 [0.06]	0.591	-0.14 [0.03]	<0.001	0.07 [0.4]	0.057
	Sex: Male versus Female	-0.45 [1.30]	0.731	1.82 [0.78]	0.019	-1.37 [0.90]	0.127
	Live in couple: Yes versus No	1.65 [1.25]	0.187	1.51 [0.75]	0.043	0.25 [0.87]	0.770
	Have children: Yes versus No	0.06 [1.14]	0.960	0.58 [0.69]	0.398	0.30 [0.79]	0.704

Professional characteristics	ED						
	Marseille versus Avignon	2.32 [1.33]	0.246	3.02 [0.78]	<0.001	-1.97 [0.91]	0.095
	Marseille versus Montpellier	2.03 [1.42]	0.458	1.03 [0.85]	0.223	0.14 [0.99]	1.000
	ED professional categories						
	Physician versus Paramedic	4.18 [1.47]	0.014	2.7 [0.88]	0.006	-2.22 [1.03]	0.094
	Physician versus A/T	8.42 [2.13]	<0.001	5.05 [1.28]	<0.001	-0.48 [1.49]	1.000
	1^st^ job in an ED: Yes versus No	-1.29 [1.23]	0.294	-0.15 [0.74]	0.838	0.79 [0.85]	0.350
	Time working in the ED: >5 years versus ≤5 years	1.96 [1.13]	0.085	0.003 [0.68]	0.997	-0.15 [0.79]	0.851
	Work rhythm: Night or Day/Night versus Day	2.66 [1.16]	0.022	1.84 [0.69]	0.008	-2.02 [0.79]	0.012
	Job strain: Yes versus No	8.33 [1.16]	<0.001	2.67 [* *0.7]	<0.001	-3.58 [* *0.83]	<0.001
	Wish to quit the ED: Yes versus No	12.23 [1.44]	<0.001	4.26 [0.91]	<0.001	-4.17 [1.02]	<0.001

work-related health	≥1 sick leave during the last year: Yes versus No	4.14 [1.15]	<0.001	0.13 [0.69]	0.847	-1.38 [0.79]	0.082
	Sleep disorders: Yes versus No	8.09 [1.10]	<0.001	2.15 [0.69]	0.002	-1.80 [0.81]	0.026

Lifestyle	Regular sport activity: Yes versus No	-2.05 [1.16]	0.077	-0.41 [0.69]	0.558	-0.07 [0.81]	0.935
	Smoker: Yes versus No	-0.82 [1.17]	0.486	0.15 [0.70]	0.828	1.21 [0.81]	0.136
	> 4 cups of coffee per day: Yes versus No	0.14 [1.47]	0.923	0.16 [0.88]	0.852	0.26 [1.01]	0.799
	Daily consumption of alcohol: Yes versus No	-4.95 [6.45]	0.444	1.77 [3.84]	0.645	-0.002 [0.86]	0.998

Quality of life	MCS	-0.69 [0.04]	<0.001	-0.23 [0.03]	<0.001	0.25 [0.04]	<0.001
	PCS	-0.19 [0.09]	0.043	0.07 [0.05]	0.165	0.005 [0.06]	0.938

SD: standard deviation.

**Table 3:** Multivariate analysis of covariance of factors associated with MBI emotional exhaustion, depersonalization, and personal achievement scores.

**Category**	**Covariate**	**EE**		**DP**		**PA**	

		Coefficient [SD]	p	Coefficient [SD]	p	Coefficient [SD]	p

Demographics	Age (years)	-		-0.13 [0.03]	<0.001	-	
	Sex: Male versus Female	-		1.63 [0.75]	0.029	-	
	Live in couple: Yes versus No	-		1.23 [0.70]	0.079	-	

Professional characteristics	ED						
	Marseille versus Avignon	-		3.12 [0.74]	<0.001	-	
	Marseille versus Montpellier	-		1.79 [0.81]	0.080	-	
	ED professional categories						
	Physician versus Paramedic	3.85 [1.12]	0.002	1.98 [0.86]	0.066	-	
	Physician versus A/T	6.15 [1.63]	0.001	3.13 [1.24]	0.036	-	
	Work rythm: Night or Day/Night versus Day	-		-		-1.54 [0.76]	0.042
	Job strain: Yes versus No	3.36 [0.96]	0.001	1.96 [0.73]	0.008	-2.27 [0.84]	0.007

work-related health	≥1 sick leave during the last year: Yes versus No	1.69 [0.87]	0.052	-		-	
	Sleep disorders: Yes versus No	3.49 [0.87]	<0.001	-		-	

Quality of life	Mental component summary score	-0.53 [0.05]	<0.001	-0.14 [0.03]	<0.001	0.21 [0.04]	<0.001
	Physical component summary score	-0.36 [0.7]	<0.001	-		-	

SD: standard deviation.

## Data Availability

The data collected by questionnaire and used to support the conclusions of this study are available upon request from the corresponding author.

## References

[1] Khireddine-Medouni I., Lemaître A., Homère J. (2015). La souffrance psychique en lien avec le travail chez les salariés actifs en France entre 2007 et 2012, à partir du programme MCP. *Bulletin Épidémiologique Hebdomadaire*.

[2] Bureau international du Travail (1993). Le travail dans le monde. *Chapitre 5: Le stress dans le monde du travail*.

[3] International Labour Organization Workplace stress: A collective challenge. World day for safety and health at work. http://www.ilo.org/wcmsp5/groups/public/-ed_protect/-protrav/-safework/documents/publication/wcms_466547.pdf.

[4] Leka S., Griffiths A., Cox T. (2004). Organisation du travail et stress. Institute of work, health & organisations. *Série protection de la santé des travailleurs n°3*.

[5] International Labour Office Stress prevention at work checkpoints: Practical improvements for stress prevention in the workplace. http://www.ilo.org/wcmsp5/groups/public/-ed_protect/-protrav/-safework/documents/instructionalmaterial/wcms_177108.pdf.

[6] Lemaitre A., Valenty M. (2014). Programme de surveillance des maladies à caractère professionnel (MCP) en France. *Résultats des Quinzaines MCP 2008 à 2011*.

[7] Eurofound, EU-OSHA. Psychosocial risks in Europe: Prevalence and strategies for prevention. http://irep.ntu.ac.uk/id/eprint/31136/1/PubSub8692_Hassard.pdf.

[8] Hirigoyen M. F. (1998). Le harcèlement moral: la violence perverse au quotidien. *La Découverte & Syros*.

[9] Nasse P., Legeron P. Rapport sur la détermination, la mesure et le suivi des risques psychosociaux au travail. http://www.dgdr.cnrs.fr/drh/protect-soc/documents/fiches_rps/rapport_légeron.pdf.

[10] (2015). Plans de santé au travail (PST3) 2016-2020. *Ministère du Travail*.

[11] Repérage et prise en charge cliniques du syndrome d’épuisement professionnel ou burnout. https://www.has-sante.fr/portail/upload/docs/application/pdf/2017-05/dir56/rapport_elaboration_burnout.pdf.

[12] Haute Autorité de Santé - Revue de littérature: qualité de vie au travail et qualité des soins. https://www.has-sante.fr/portail/jcms/c_2610262/fr/revue-de-litterature-sur-qualite-de-vie-au-travail-et-qualite-des-soins.

[13] Olié J-P., Légeron P. (2016). Le burnout. *Académie Nationale de Médecine*.

[14] Arnaudo B., Léonard M., Sandret N., Cavet M., Coutrot T., Rivalin R. (2013). Les risques professionnels en 2010: de fortes différences d’exposition selon les secteurs. *INRS*.

[15] Shanafelt T. D., Boone S., Tan L. (2012). Burnout and satisfaction with work-life balance among US physicians relative to the general US population. *JAMA Internal Medicine*.

[16] Durand A.-C., Gentile S., Devictor B. (2011). ED patients: how nonurgent are they? Systematic review of the emergency medicine literature. *The American Journal of Emergency Medicine*.

[17] Braun F. (2012). Urgentistes au bord de la crise de nerf. *Annales Françaises de Médecine D'urgence*.

[18] Lala A. I., Sturzu L. M., Picard J. P., Druot F., Grama F., Bobirnac G. (2016). Coping behavior and risk and resilience stress factors in French regional emergency medicine unit workers: a cross-sectional survey. *Journal of Medicine and Life*.

[19] Laurent A., Chahraoui K., Carli P. (2007). Les répercussions psychologiques des interventions médicales urgentes sur le personnel SAMU. Étude portant sur 50 intervenants SAMU. *Annales Médico-psychologiques, revue psychiatrique*.

[20] Laurent A., Chahraoui K. (2012). L’impact du stress professionnel sur les intervenants SMUR. *Pratiques Psychologiques*.

[21] Adriaenssens J., De Gucht V., Maes S. (2015). Determinants and prevalence of burnout in emergency nurses: A systematic review of 25 years of research. *International Journal of Nursing Studies*.

[22] Sende J., Jbeili C., Schvahn S. (2012). Facteurs de stress et conséquences du stress en médecine d’urgence: enquête nationale. *Annales françaises de médecine d'urgence*.

[23] Bellagamba G., Gionta G., Senergue J., Bèque C., Lehucher-Michel M.-P. (2015). Organizational factors impacting job strain and mental quality of life in emergency and critical care units. *International Journal of Occupational Medicine and Environmental Health*.

[24] Trousselard M., Dutheil F., Naughton G. (2016). Stress among nurses working in emergency, anesthesiology and intensive care units depends on qualification: a Job Demand-Control survey. *International Archives of Occupational and Environmental Health*.

[25] Loi n° 2004-801 du 6 août 2004 relative à la protection des personnes physiques à l'égard des traitements de données à caractère personnel et modifiant la loi n° 78-17 du 6 janvier 1978 relative à l'informatique, aux fichiers et aux libertés. https://www.legifrance.gouv.fr/affichTexte.do?cidTexte=JORFTEXT000000441676.

[26] Niedhammer I., Ganem V., Gendrey L., David S., Degioanni S. (2006). Propriétés psychométriques de la version française des échelles de la demande psychologique, de la latitude décisionnelle et du soutien social du 'Job Content Questionnaire' de Karasek: résultats de l'enquête nationale SUMER. *Santé Publique*.

[27] Gandek B., Ware J. E., Aaronson N. K. (1998). Tests of data quality, scaling assumptions, and reliability of the SF-36 in eleven countries: results from the IQOLA Project. *Journal of Clinical Epidemiology*.

[28] Ware J. E., Kosinski M., Keller S. D. (1995). *How to Score the SF-12 Physical and Mental Health Summary Scales*.

[29] Jenkinson C., Layte R., Jenkinson D. (1997). A shorter form health survey: can the SF-12 replicate results from the SF-36 in longitudinal studies?. *Journal of Public Health*.

[30] Maslach C., Jackson S., Leiter M. (1997). The maslach burnout inventory manual. *Evaluating Stress: A Book of Resources*.

[31] Maslach C., Jackson S. E., Leiter M. P. (1996). *Maslach Burnout Inventory Manual*.

[32] Ben-Itzhak S., Dvash J., Maor M., Rosenberg N., Halpern P. (2015). Sense of meaning as a predictor of burnout in emergency physicians in Israel: a national survey. *Clinical and Experimental Emergency Medicine*.

[33] Escribà-Agüir V., Martín-Baena D., Pérez-Hoyos S. (2006). Psychosocial work environment and burnout among emergency medical and nursing staff. *International Archives of Occupational and Environmental Health*.

[34] Tarcan M., Hikmet N., Schooley B., Top M., Tarcan G. Y. (2017). An analysis of the relationship between burnout, socio-demographic and workplace factors and job satisfaction among emergency department health professionals. *Applied Nursing Research*.

[35] Schooley B., Hikmet N., Tarcan M., Yorgancioglu G. (2016). Comparing burnout across emergency physicians, nurses, technicians, and health information technicians working for the same organization. *Medicine*.

[36] Howlett M., Doody K., Murray J., LeBlanc-Duchin D., Fraser J., Atkinson P. R. (2015). Burnout in emergency department healthcare professionals is associated with coping style: A cross-sectional survey. *Emergency Medicine Journal*.

[37] Jalili M., Sadeghipour Roodsari G., Bassir Nia A. (2013). Burnout and associated factors among iranian emergency medicine practitioners. *Iranian Journal of Public Health*.

[38] Lloyd S., Streiner D., Shannon S. (1994). Burnout, depression, life and job satisfaction among Canadian emergency physicians. *The Journal of Emergency Medicine*.

[39] Gillespie M., Melby V. (2003). Burnout among nursing staff in accident and emergency and acute medicine: A comparative study. *Journal of Clinical Nursing*.

[40] Hooper C., Craig J., Janvrin D. R., Wetsel M. A., Reimels E. (2010). Compassion satisfaction, burnout, and compassion fatigue among emergency nurses compared with nurses in other selected inpatient specialties. *Journal of Emergency Nursing*.

[41] Kuhn G., Goldberg R., Compton S. (2009). Tolerance for uncertainty, burnout, and satisfaction with the career of emergency medicine. *Annals of Emergency Medicine*.

[42] Estryn-Behar M., Doppia M.-A., Guetarni K. (2011). Emergency physicians accumulate more stress factors than other physicianse - Results from the French SESMAT study. *Emergency Medicine Journal*.

[43] Goldberg R., Boss R. W., Chan L. (1996). Burnout and its correlates in emergency physicians: Four years' experience with a wellness booth. *Academic Emergency Medicine*.

[44] La L. P. (2018). D’Agnès Buzyn dans l’état de crise actuel des urgences. *Hospimedia*.

[45] Libot L. (2018). On ne peut plus se permettre le luxe d’avoir de 'l’open bar' aux urgences 24h/24 quand on veut. *Hospimedia*.

[46] Galam É. (2007). Burn out des médecins libéraux - 1re partie: une pathologie de la relation d’aide. *Médecine*.

[47] He M., Wang Q., Zhu S. (2012). Health-related quality of life of doctors and nurses in China: findings based on the latest open-access data. *Quality of Life Research*.

[48] Silva A. A., Souza J. M., Borges F. N., Fischer F. M. (2010). Health-related quality of life and working conditions among nursing providers. *Revista de Saúde Pública*.

[49] Bovier P. A., Arigoni F., Schneider M., Gallacchi M. B. (2009). Relationships between work satisfaction, emotional exhaustion and mental health among Swiss primary care physicians. *European Journal of Public Health*.

[50] Lesuffleur T., Chastang J.-F., Sandret N., Niedhammer I. (2015). Psychosocial factors at work and occupational injury: Results from the French National SUMER Survey. *Journal of Occupational and Environmental Medicine*.

[51] Arnaudo B., Léonard M., Sandret N., Cavet M., Coutrot T., Rivalin R. (2013). Les risques professionnels en 2010: de fortes différences dexposition selon les secteurs. *INRS*.

[52] Loquet J., Ricroch L. (2016). Les conditions de travail dans les établissements de santé. *Panoramas*.

[53] Westbrook J. I., Raban M. Z., Walter S. R., Douglas H. (2018). Task errors by emergency physicians are associated with interruptions, multitasking, fatigue and working memory capacity: a prospective, direct observation study. *BMJ Quality & Safety*.

[54] Wallace J. E., Lemaire J. B., Ghali W. A. (2009). Physician wellness: a missing quality indicator. *The Lancet*.

